# Assessment of Executive Functions in Methamphetamine-addicted Individuals: Emphasis on Duration of Addiction and Abstinence

**DOI:** 10.18869/nirp.bcn.8.2.147

**Published:** 2017

**Authors:** Majid Farhadian, Malahat Akbarfahimi, Peyman Hassani Abharian, Seyedeh Golaleh Hosseini, Susan Shokri

**Affiliations:** 1. Department of Occupational Therapy, School of Rehabilitation Sciences, Iran University of Medical Sciences, Tehran, Iran.; 2. Department of Cognitive Rehabilitation, Institute for Cognitive Sciences Studies (ICSS), Tehran, Iran.

**Keywords:** Addiction, Abstinence, Methamphetamine, Executive function, Assessment

## Abstract

**Introduction:**

Several studies have conducted on impairments of executive functions in individuals with methamphetamine addiction; however, only a few have investigated the relationship between executive functions and duration of addiction or abstinence. This study was designed to assess the executive functions in methamphetamine-addicted individuals in relation to the duration of addiction or abstinence.

**Methods:**

A total of 161 subjects aged between 20 and 45 years were categorized into three subgroups: currently abusing (n=41), abstinent (n=60), and control healthy individuals (n=60). A battery of standardized executive function tasks, including Stroop test, Wisconsin Card Sorting test, and Tower of London task, were administered. Data were analyzed using Pearson correlation coefficient, analysis of variance, and post hoc Bonferroni test with SPSS16.0.

**Results:**

Methamphetamine-addicted and abstinent subjects performed worse than the controls. Methamphetamine-abstinent subjects performed better than the currently methamphetamine abusers in most executive functions. Duration of addiction and abstinence were correlated with executive dysfunctions.

**Conclusion:**

This study revealed that although executive functions may be improved by protracted abstinence, executive dysfunctions are not completely relieved, and specific attention to planning and implementation of intervention programs are necessary.

## 1. Introduction

Methamphetamine (METH) is an addictive psychostimulant drug, which is one of the most widely abused drugs all over the world. METH addiction associates with negative medical, psychiatric, and behavioral consequences, which could meddle with psychosocial functions and daily living skills, and may result in disability (Scott et al., 2007). METH addiction may lead to profound cognitive deficits (Salo et al., 2002), including deficits in executive functions, verbal memory, processing speed, response inhibition, and attention tasks (Meredith, Jaffe, Ang-Lee, & Saxon, 2005; Scott et al., 2007). Out of these cognitive impairments, executive functions are most commonly impaired in METH-addicted individuals (Scott et al., 2007). “Executive function” is a comprehensive term, including a wide range of cognitive processes and behavioral competencies. It also consists of verbal reasoning, problem solving, planning, sequencing, focusing, multitasking, cognitive flexibility, and dealing with novelty (Burgess, Veitch, de Lacy Costello, & Shallice, 2000; Damasio, 1995; Grafman & Litvan, 1999; Stuss, Shallice, Alexander, & Picton, 1995).

Despite the increasing number of studies investigating executive function deficits in METH-addicted individuals, there are controversies about the relationship between the duration of addiction or abstinence, and executive function performance. One study showed that executive functions may be improved during prolonged METH abstinence (Salo et al., 2009). Another study that used imaging technology and followed up METH-addicted subjects during the abstinence period showed significant recovery in cognitive task performance (Volkow et al., 2001). On the other hand, some studies reported that longer periods of abstinence may lead to more improvement in cognitive functions, although some deficits may remain, particularly cognitive inhibition (Johanson et al., 2006). Moreover to our best knowledge, the effects of METH use correlates such as length of abstinence or addiction, on executive functions have not been investigated adequately (Scott et al., 2007). Therefore, the present study was designed to compare the performance of METH-addicted and abstinent individuals in executive functions, and their relationship with duration of addiction and abstinence.

## 2. Methods

### 2.1. Participants

A total of 161 subjects participated in this study (41 currently METH abusing, 60 abstinent and 60 healthy individuals). Both the currently abusing subjects and abstinent participants were recruited through outpatient therapeutic centers that provided behavioral treatments (i.e. cognitive–behavioral therapy) and educational counseling. Inclusion criteria for currently METH abusing subjects were as follows: 1) Diagnostic and Statistical Manual of Mental Disorders, 4^th^ Edition (DSM-IV) criteria for METH addiction; 2) not using other pharmacological treatments simultaneously; 3) not being diagnosed with DSM-IV Axis I or II disorders or positive HIV; and 4) not suffering from neurological alterations (central nervous system neurological disorder, head injury with loss of consciousness, or seizure disorders) or brain scan abnormalities in their histories. Inclusion criteria for METH-abstinent individuals were all the above mentioned criteria, plus the current experience of a period of abstinence for at least 15 days (from any drug of abuse, including alcohol), so that withdrawal symptoms can be ruled out. In addition, rapid urinary tests for detection of abused drugs or their metabolites were routinely conducted for these individuals every week, allow us to rule out drug use throughout the period of abstinence. The inclusion criteria for healthy control individuals were all mentioned above, except addiction to methamphetamine, as well as no history of mental retardation or learning disability and any chronic diseases or using psychiatric or other types of medicines.

### 2.2. Procedure

This study was approved by the Ethics Committee of Iran University of Medical Sciences. All measures were administered individually by an occupational therapist in a single session that lasted about 1 hour (including the breaks). Participants were advised about the objectives, advantages, and possible inconveniences associated with the research protocol and were ensured that their participation in the research is voluntary and they could withdraw from the study whenever they wanted. After obtaining signed consent forms from the participants, the study instruments were administered to them. Following completion of assessments, all the participants received a report about their performance in the tasks.

### 2.3. Instruments

#### 2.3.1. Wisconsin Card Sorting Test (WCST)

This test is a gold standard tool for the assessment of problem solving and set shifting (Heaton, 1993). Subjects completed the computerized version of the WCST, in which they were instructed to select one of four target cards that matched a test card in shape, color, or number of stimuli. Per manual instruction, the play rules were not explained to the participants; for each trial, only the computer feedback helps the participant to find out whether he/she performed correctly or not.

#### 2.3.2 Stroop Test

Stroop test measures distortion of attention, inhibition, and set shifting (MacLeod, 1991). Participants were instructed to use computerized version of the Stroop test, which consists of three stages. In the first stage (color cards), color circles (blue, red, yellow or green) appear in the middle of the screen surface, and the subject should immediately press the same color key on the labeled Num-Lock keyboard. In the second stage (pilot stage), a word with scrambled colors appears, and the subject should press the correct color key according to the word without attention to the color of the word. Third stage is similar to the second one, but the timing of this stage is longer than the second stage.

#### 2.3.3. Tower of London Task

Tower of London Task is commonly used for assessment of executive planning (Krikorian, Bartok, & Gay, 1994). In this study, a computerized version of the Tower of London task was used to display the goal. The task starts with simultaneous display of different configurations in the left and right halves of the screen. Participants were asked to transform the start state (left field) into the goal state, following three basic rules: 1) only one ball must be moved at a time, 2) a ball may be moved only if no other ball is on top of it, and 3) three balls may be placed on the tallest peg, two balls on the middle peg, and one ball on the shortest peg. The program did not allow rule-incongruent moves. This version provided the minimal number of moves to solve the problem (cue). Twelve Tower of London task problems must be solved, in 3 to 7 moves, which are presented in an easy to hard order. The task had no time limit and the participants did not get any feedback on their success.

### 2.4. Data analysis

Data analysis was performed using SPSS 16. Based on Kolmogorov–Smirnov test, it was proved that the variables had a normal distribution. Pearson correlation coefficient and analysis of variance (ANOVA) were used to study the relationship between the variables. Significant group differences were evaluated using the post hoc Bonferroni test. All tests were performed at a confidence interval of 95% (P<0.05).

## 3. Results

One hundred and sixty-one subjects participated in this study (41currently METH abusing subjects, 60 METH abstinent subjects and 60 healthy control subjects). The samples included 22 males and 19 females in currently METH abusing group, 33 males and 27 females in METH abstinent group and 33 males and 27 females in the control group. There were no significant difference between the level of education and ages of these 3 groups (Table 1).

**Table 1.** Descriptive and METH use characteristics of this study groups (41 METH current abusers, 60 METH abstinent subjects and 60 healthy non addicted subjects).

**Group**	**Current Abusers**	**Abstinent Subjects**	**Control Subjects**
	**Mean±SD**	**Mean±SD**	**Mean±SD**
Age (Year)	35.04±4.54	34.65±5.89	33.18±6.95
Education (Year)	11.76±3.56	10.96±3.28	12.46±4.18
Age of onset (Year)	27.07±5.18	27.48±5.094	-
Duration of addiction (Month)	45.36±13.94	40.3±14.06	-
Duration of abstinence (Day)	-	46.91±12.77	-

There were significant differences between these 3 groups about their performance in executive functions (EFs). As it is shown in Table 2, the control group had better performance compared to abstinents and current abusers. Moreover, in Tower of London task that evaluated executive planning, and the category subset of WCST; abstinence group had higher scores that indicated better performance than currently abusing group. In addition, lower interference number of Stroop test, indicated better performance of abstinent individuals on this subtest compared to the currently abusers. There was no statistically significant differences between these two groups about presentative error subset of WCST and the interference time subset of the stroop test (Table 3).

**Table 2.** Mean scores and standard deviations and ANOVA results for currently METH-abusers, abstinent subjects and healthy non-addicted individuals in executive function tests.

**Assessment**	**Current Abusers (n=41)**	**Abstinent Subjects (n=60)**	**Healthy Subjects (n=60)**	**ANOVA**	
				**F**	**P**
ToL	19.39±2.84	21.58±4.23	32.18±3.33	197.43	0.000
WCST (persenative error)	11.73±7.15	10.13±6.47	3.73±3.06	30.07	0.000
WCST (categories)	1.70±0.95	2.46±1.35	4.5±1.41	70.43	0.000
ST (Interference time)	116.85±68.82	100.58±156.42	50.55±45.21	3.38	0.000
ST (Interference number)	14.07±3.50	12.25±4.92	1.91±2.16	199.69	0.037

ToL: Tower of London;

WCST: Wisconsin Card Sorting Test,

ST: Stroop Test.

**Table 3.** Differences between abstinence, currently abusing and control groups in executive function taks using post-hoc Bonferroni test.

**Dependent Variable**	**Groups**		**Mean Difference**	**Std. Error**	**95% Confidence Interval**	
					**Upper Bound**	**Lower Bound**
TOL	Normal	Abstinence	10.60*	0.65	9.01	12.1856
	Normal	Currently using	12.79*	0.72	11.03	14.5528
	Currently using	Abstinence	−2.19*	0.78	−3.9528	−0.4333
WCST (Categories)	Normal	Abstinence	2.11*	0.23	1.5456	2.6877
	Normal	Currently using	2.87*	0.26	2.2422	3.5098
	Currently using	Abstinence	−.75*	0.29	−1.3931	−0.1256
WCST (Persenative error)	Normal	Abstinence	−6.40*	1.03	−8.9042	−3.8958
	Normal	Currently using	−7.99*	1.14	−10.77	−5.2191
	Currently using	Abstinence	1.59	1.19	−1.1809	4.3776
ST (Interference number)	Normal	Abstinence	−11.33*	0.68	−12.98	−9.6838
	Normal	Currently using	−13.15*	0.75	−14.98	−11.3259
	Currently using	Abstinence	1.82	0.81	−0.0075	3.6538
ST (Interference time)	Normal	Abstinence	−50.03	25.04	−110.62	10.5626
	Normal	Currently using	−66.30	27.79	−133.55	0.9471
	Currently using	Abstinence	16.27	27.37	−50.98	83.5211

ToL: Tower of London;

WCST: Wisconsin Card Sorting Test,

ST: Stroop Test.

* The mean difference is significant at the 0.05 level.

As it is shown in Table 4, there are significant relationships between duration of addiction and all neuropsychological tests, except the interference number subset of the stroop test in currently abusing subjects. Nevertheless, only two tasks were in relationship with duration of abstinent, which were TOL and category subset of WCST.

**Table 4.** Relationship of neuropsychological tests and methamphetamine usage status in currently abusing and abstinent subjects.

	**ToL**	**WCST (Perserverative Error)**	**WCST (Categories)**	**ST (Interference Time)**	**ST (InterferenceNumber)**
Duration of addiction (Currently abusers subjects) Level of significance (P-value)	−0.748(0.000)	0.752(0.000)	−0.499(0.001)	0.422(0.006)	0.176(0.272)
Duration of abstinent (Abstinent subjects) Level of significance (P-value)	0.299(0.02)	0.18(0.091)	0.273(0.035)	0.246(0.058)	−0.133(0.310)

ToL: Tower of London;

WCST: Wisconsin Card Sorting Test,

ST: Stroop Test.

## 4. Discussion

The present study examined the executive functions and their relationships with the duration of drug addiction and abstinence in currently METH abusing and abstinent subjects, in comparison with healthy non-drug-taking individuals. The results indicated that currently abusing and abstinent groups performed significantly worse than the control group in their executive functions.

These results are consistent with those of previous studies, which suggested greater executive impairments in METH addicts compared with healthy individuals (Panenka et al., 2013; Weber et al., 2012). However, METH-abstinent group showed better performance in executive function tests than currently abusing subjects. It appears that the abstinence period led to improvement in executive functions such as problem solving and executive planning. This finding is supported by other studies reporting that METH-addicted individuals perform better in executive function tests when tested in the early abstinence phase (Jaffe et al., 2005; Volkow et al., 2001; Wang et al., 2014).

A comparison of performance between the groups revealed attentional set shifting impairment even after the early abstinence phase. This finding was consistent with other studies reported that METH addiction has more attentional problems compared with other drug addictions that cause significant impairments in some executive functions related to maintaining attention and resisting irrelevant stimuli (Hekmat, Alam Mehrjerdi, Moradi, Ekhtiari, & Bakhshi, 2011; Hosak et al., 2012). METH addiction causes deficits in frontal and cingulate brain regions, which are essential for cognitive control (Jan et al., 2014). Furthermore, perseverative errors in WCST are also related to frontal lobe functions. Altogether, the findings of this study suggest that impairments in frontal lobe functions remain intact during the early phase of abstinence, and it appears that some executive function impairments need a prolonged abstinence period for an optimal recovery.

The study findings also indicated that among METH currently abusing subjects, individuals who reported a longer duration of addiction performed worse than individuals with a shorter duration of addiction. Few studies have reported that METH-addicted individuals performed worse on cognitive tests during a longer time of addiction. A study that investigated cognitive control and drug abstinence in terms of the relationship between the duration of METH addiction and executive function tasks reported that longer years of addiction would result in worse performance in Stroop task (Salo et al., 2009). Another study had also shown the inverse relationship between addiction duration and cognitive flexibility, attention, and speed of mental processing in METH-abstinent individuals (Hekmat et al., 2011). It appears that the longer duration of METH addiction causes more impairments in executive function tasks and frontal lobe functions, which are primarily engaged in executive functions.

The better executive function performance during longer abstinence, as observed in this study, is in agreement with previous studies. An earlier study that used the Stroop test showed that METH-addicted individuals who had been abstinent for 3 weeks to 6 months, performed worse than METH-abstinent individuals for at least 1 year (Salo et al., 2009). METH-addicted individuals who were abstinent for longer time showed better performance on WCST, although there was no significant difference between the groups on the Stroop test and trail-making performance (Kim et al., 2006). Therefore, there is a tendency toward cognitive improvement in the early abstinence period of time, although METH-addicted individuals need prolonged abstinence time for optimal executive function improvement.

Nevertheless, this study suggests that METH use could cause a decline in executive functions that might persist for a long time even after abstinence. In addition, impairments in executive functions, not only affect the patient’s daily life, but also influence the management of their addiction. Therefore, it is necessary to consider a comprehensive assessment of executive functions through various neuropsychological tests in METH-addicted individuals, after which the addiction management team should consider these evaluations as an essential part of planning for therapy and rehabilitation process.

### 4.1. Limitations

There were several potential limitations in the present study. First, it included a small number of subjects recruited from treatment facilities rather than from a generalized community sample. Second, moderating variables such as the frequency of METH use and total cumulative dose may affect the cognitive performance of METH abusers. This study evaluated three separate groups of subjects with no clear data on these moderating variables. Therefore, longitudinal studies of METH abusers are warranted to determine the long-term effects of METH abuse and abstinence.
